# Supplementary material for: MetaRibo-Seq measures translation in microbiomes
Source: Nat Commun. 2020 Jun 29;11:3268. doi: 10.1038/s41467-020-17081-z (PMC7324362; doi:10.1038/s41467-020-17081-z)
Supplement: Supplementary file 10 — Supplementary Data 7 [file 41467_2020_17081_MOESM10_ESM.zip › File2/Confidence_High_Taxonomy/206919_out.krona.html]

Javascript must be enabled to view this page.

members
magnitude
magnitudeUnassigned
count
unassigned
taxon
rank

206919\_out

2

2
superkingdom
2

976
2
phylum

200643
2
class

2
order
171549

815
family
2

1

SRS054059\_contig\_number\_contig-100\_3611.38964
816
genus
2

820

SRS019601\_contig\_number\_29399
1
species
